# The effectiveness of internet-delivered cognitive behavioural therapy for those with bulimic symptoms: a systematic review: A review of iCBT treatment for bulimic symptoms

**DOI:** 10.1186/s13104-018-3843-2

**Published:** 2018-10-22

**Authors:** Alexandra Pittock, Laura Hodges, Stephen M. Lawrie

**Affiliations:** Division of Psychiatry, Royal Edinburgh Hospital, University of Edinburgh, Edinburgh, EH10 5HF UK

**Keywords:** Eating disorders, Cognitive therapy, Treatment, Bulimia nervosa, Internet

## Abstract

**Objective:**

This review looked at internet-delivered cognitive behavioural therapy (iCBT) as a possible treatment for patients with bulimic symptoms. CBT has been established as an effective treatment; however, waiting lists lead to delayed initiation of treatment. iCBT is a possible delivery method to combat this. Medline, EMBASE and PsycInfo were searched for controlled trials using iCBT as a treatment for patients with bulimia nervosa (BN), subthreshold BN or ‘eating disorders not otherwise specified’ with bulimic characteristics (EDNOS-BN). The literature search returned 482 papers. 5 met the review criteria and were compared in characteristics, methodological quality and outcomes. Outcomes were analysed by calculation of effect sizes; iCBT was evaluated on reduction in binge eating and purging post treatment and at follow-up.

**Results:**

Participants were mostly female with an average age range of 23.7–31 years. 4 studies demonstrated good methodological quality. 1 did not report all of the outcome data, increasing the likelihood of bias. Only 1 study showed widespread benefit over waiting list controls. iCBT was shown to reduce behaviours but was not found to be superior to bibliotherapy or waiting list. Further large-scale studies are required to make conclusive recommendations.

**Electronic supplementary material:**

The online version of this article (10.1186/s13104-018-3843-2) contains supplementary material, which is available to authorized users.

## Introduction

Bulimia nervosa (BN) is described by DSM-5 as frequent episodes of binge eating followed by compensatory behaviours such as self-induced vomiting to avoid gaining weight [1]. It is approximately three times more common in women, with lifetime prevalence estimated at 1.5% and point prevalence at 0.5%. Women under thirty have the highest risk of developing the disease [2]. Cognitive behavioural therapy (CBT) is an accepted form of treatment; [3] however funding limitations can lead to waiting lists. One proposal to address this is delivering CBT via another medium. A systematic review by Polnay et al. noted that 25% of patients with BN are offered group CBT, which was more effective than no treatment, but were unable to determine any differences between group and individual CBT [4].

Internet-delivered CBT (iCBT) is another alternative. Andersson et al. reviewed iCBT vs. face-to-face CBT for psychiatric disorders and found that, whilst the research supported equivalence, it was insufficient to draw definitive conclusions [5]. Loucas et al. conducted a meta-analysis of internet therapies for eating disorders. They noted that study heterogeneity made it difficult to assess efficacy [6]. Fairburn and Murphy looked specifically at iCBT for patients with binge eating; they found it was acceptable to female patients and some made a significant improvement [7].

Given BN’s prevalence we felt it important into focus on it specifically. CBT has been shown to be more effective for BN than other types of eating disorder and is recommended by NICE [8]. Research has also shown a higher placebo response and greater remittance rates in studies with binge eating disorder (BED) populations [9]. Thus inclusion of this patient group could skew results. This review aims to establish whether iCBT is effective for adults with BN and subthreshold presentations. This will be evaluated primarily as a reduction in binge eating and purging.

## Main text

### Inclusion criteria

Papers were considered eligible if they focused on participants with BN, subthreshold BN or EDNOS-BN; evaluated iCBT treatment; were randomised or clinical controlled trials and reported outcomes quantitatively.

### Exclusion criteria

Papers were excluded if they focused on undifferentiated eating disorders, lacked controls, studied solely dropout rates, focused on adolescents, included participants with BED or on antidepressants. Due to the authors’ linguistic abilities papers were only considered if they were in English, French or Italian. There was no exclusion based on age of paper since the advent of internet-based therapies is recent.

### Study selection

We searched EMBASE, Medline and PsycInfo from date of inception using the algorithm: (exp bulimia nervosa/OR bulimia OR eating disorders) AND (exp Randomised controlled trials/OR random) AND (exp Cognitive behavioural therapy/OR self help OR computerised CBT OR internet CBT). Search performed: 8th April 2018; 481 papers returned.

**Fig. 1:**
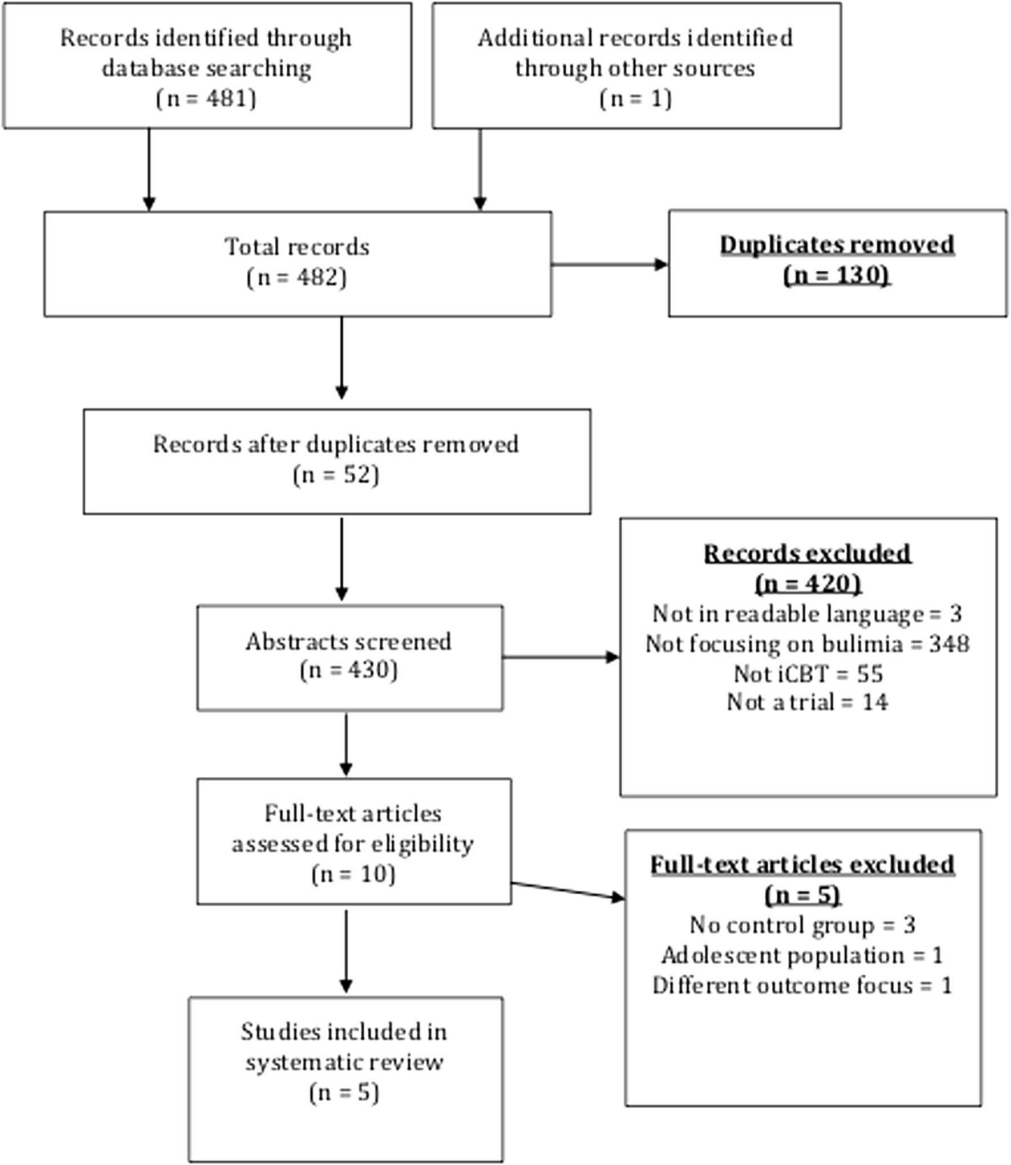
PRISMA flow-chart of study selection [10]

Two reviewers examined titles and abstracts independently. This included articles listed as accepted but as yet unpublished. Relevant papers and review articles’ references were also searched. Duplicates were excluded. Selection of papers was discussed with all three authors to prevent omission of relevant studies. Ten articles met inclusion criteria. The study selection is detailed below in Fig. 1.

### Assessment

We compared study characteristics and assessed methodological quality using the risk of bias tool developed by the Cochrane Collaboration. This encompasses five areas of bias: selection, performance, detection, attrition and reporting. Possible sources of bias are identified, and assessors are asked to describe how papers sought to prevent these from occurring. Finally risk of bias is judged as ‘low’, ‘unclear’ or ‘high’ [11]. Fernández-Aranda et al. exhibited potential for bias due to incomplete outcome reporting [12]. Their research was partly funded by the intervention manufacturer. Three studies evidenced robust study designs but their results were potentially biased [13–15]. Sánchez-Ortiz’s results were likely less relevant due to focusing solely on female university students. Ruwaard et al. relied solely on self-reported outcome measures [14, 15]. Wagner et al. had a low risk of bias overall but failed to acknowledge whether they included dropout data in their final analysis [16].

Our assessment is detailed in Additional file 1: Table S1.

### Statistical analysis

We evaluated iCBT on reduction in binge eating, self-induced vomiting and purging post-treatment and at follow-up. Effect sizes were calculated using means and standard deviations, where given. These were expressed as a standardised mean difference (SMD), corrected using Hedges’ g for small sample size to reduce positive bias, with a 95% confidence interval (CI). One study did not provide raw data for SMDs but published Cohen’s d effect sizes instead, which we converted to Hedges’ g [12]. SMDs were then classified into level of effect as follows: < 0.2—very small, 0.2—small, 0.5—medium, and > 0.8—large. A meta-analysis was not conducted due to the heterogeneity of studies—in intervention length, controls and follow-up periods—meaning most SMDs were not directly comparable.

### Characteristics

The studies are summarised in Additional file 2: Table S2 and Additional file 3: Table S3. All were published in English, used iCBT as their intervention and included patients with diagnoses of EDNOS-BN and/or BN [12–16]. Wagner et al. also included patients with bulimic symptomatology, which was poorly defined [16]. Fernández-Aranda et al. did a controlled study; [12] the rest were RCTs. All studies had follow-up ranging from 6 to 18 months [12–16].

Sample sizes ranged from 62 to 196 with an average age of 23.7 to 31 years. Only Zerwas et al. included male patients and commented on ethnic diversity [13]. All studies used different iCBT programmes supported by therapists, varying between 2 and 7 months. WLT participants received the intervention at the end of the study. Two studies included comparison between iCBT and bibliotherapy [15, 16]. Zerwas et al. compared iCBT to face-to-face CBT (CBTF2F), using the same programme [13].

Dropout rates averaged c. 34%. Bibliotherapy had higher rates both during and after treatment [15, 16]. WLT controls had similar dropout rates [12, 14, 15]. The lowest rate was 8% at follow-up for iCBT, reported by Sánchez-Ortiz et al. [14]. The highest was 49% at follow-up for CBTF2F by Zerwas et al. [13].

### Outcomes

Additional file 3: Table S3 shows SMDs for main outcomes between treatment and controls [12].

#### Binge eating and purging

All studies assessed binge eating and purging. Large effects were reported by three studies in binge eating and purging reduction within their iCBT groups. These were sustained at follow-up but there was no overall significant difference in comparison to their controls (WLT, CBTF2F) [13–15]. Only Ruwaard et al. found iCBT to be superior to bibliotherapy and WLT in reducing purging post treatment. This was not sustained at follow-up [15]. Self-induced vomiting was also reported in three studies. Sanchez-Ortiz et al. and Wagner et al. noted moderate improvements in their iCBT group at follow-up. Only Fernàndez-Aranda et al. reported any superiority to controls, with moderate improvement post-treatment in their iCBT group, when compared to WLT [12, 14, 16].

#### Other behavioural outcomes

All studies used rating scales. Four used the Eating Disorders Examination (EDE) [13–15] or Eating Disorders Inventory (EDI) [12]. Three found high levels of improvement within their iCBT group, which were sustained at follow-up [13–15]. Only Sánchez-Ortiz et al. reported differences between scores in the iCBT group and controls; they found a large effect on EDE scores with iCBT [14]. Zerwas et al. noted there was no difference between their iCBT and CBTF2F groups but CBTF2F patients found therapy more acceptable [13]. Other rating scales used all showed small improvements in iCBT groups but this was not superior to controls [12–16].

#### Abstinence

Most studies defined abstinence as absence of a mixture of symptoms for between 1 and 3 months. All focused on the absence of binge eating, self-induced vomiting, purging/laxative use or compensatory behaviours. Rates varied with 22.1 to 46.5% being abstinent from binge eating ± other behaviours at follow-up. iCBT was significantly superior to WLT. Only Wagner et al. and Zerwas et al. did not compare iCBT to WLT. iCBT was not superior to bibliotherapy or CBTF2F but, at 18-month follow-up, abstinence rates in Wagner et al.’s study had more than halved [16]. Zerwas et al. initially found iCBT to be inferior to CBTF2F post-treatment; however there was no difference at 12-month follow-up [13].

### Stability of results

Four studies showed improvement within the iCBT group between post-treatment figures and follow-up in binge eating, purging or self-induced vomiting [13–16]. Three studies reported transient decreases in at least one behaviour when compared to their control groups post-treatment. None of these effects was sustained at follow-up [12, 13, 15]. Only Sánchez-Ortiz et al. found significant improvements in purging, EDE scores and subscales between their intervention and control groups. These showed stability at 6-month follow-up [14].

## Limitations

This review was considered as a specific follow-up to recent systematic reviews of internet therapies in eating disorders. Different eating disorders respond differently to therapies, rendering comparison difficult; patients with BN or EDNOS-BN are known to respond well to CBT, making iCBT a potential alternative [8]. Thus we explored the efficacy of iCBT for patients with BN or bulimic symptoms, aged 16 or over, based on results from controlled trials.

This focus limited the number of studies available. Five studies qualified for review. All used different iCBT programmes, developed from previous research or CBT manuals. Participants were supported by therapists although the frequency of interaction varied. All found statistically significant reductions in behaviours in their iCBT group. Only Sánchez-Ortiz et al. found any significant difference between iCBT and WLT [14]. iCBT was shown to be somewhat effective overall in reducing behaviours; however it was not significantly better than WLT.

Control groups also varied. Two included bibliotherapy, three used WLT and Zerwas et al. compared iCBT with CBTF2F. It is worth noting that research has previously shown that WLT is an inadequate control. In 2016, Cuijpers et al. reported effect sizes for CBT were higher in studies using WLT controls; they posited that WLT acts as a nocebo, increasing the CBT treatment effect [17]. Ruwaard et al. compared iCBT with bibliotherapy and WLT. Smaller effect sizes between iCBT and bibliotherapy were found than between iCBT and WLT; neither was statistically significant [15].

Definition of abstinence differed in all studies; however, the rates in iCBT were similar, at 20–30% for follow-up under 1 year. Fernández-Aranda et al. alone showed a difference between iCBT and WLT. Remission rates were also reported in three studies. Sánchez-Ortiz et al. and Wagner et al. found higher rates in iCBT than WLT [14, 16]. Varied definitions hindered comparison; however remission rates suggest that those with BN showed some improvement with iCBT, which increased as patients progressed through follow-up.

Critically no study mentioned any negative aspects of iCBT. In 2016, Crawford et al. found 0.5% of patients reported long term negative effects post therapy. They recommended informing patients of possible negative effects and monitoring these post-treatment [18]. All our studies used a variety of questionnaires pre and post intervention; none of them discussed or acknowledged any negative outcomes. Only Zerwas et al. commented on treatment acceptability post treatment and noted that CBTF2F was better tolerated than iCBT [13].

This lack of acknowledgement might explain dropout rates. Previous studies that looked at CBT for BN have reported high dropout rates [9] and this was also our finding. Overall c.30% of participants failed to complete iCBT. Sánchez-Ortiz et al. had fewer dropouts but a shorter programme and different study population [14]. Dropout rates were overall higher in WLT and bibliotherapy groups [14–16].

Sánchez-Ortiz et al. alone demonstrated significant differences between WLT and iCBT [14]. It is unlikely that Sánchez-Ortiz’s results were due to their iCBT programme, given its brevity and similarity to other studies’ interventions. One obvious difference is their study population. All patients were undergraduates. It could be hypothesised that hard-working, intelligent individuals might be more motivated to engage with treatment. Sánchez-Ortiz also demonstrated lower overall dropout rates, with 5% dropout in iCBT [14].

In conclusion there is a lack of evidence to show that iCBT has positive effects on disordered eating behaviours. Despite its popularity and recommendation by NICE, we could only find five published iCBT studies that were eligible for review. None showed clear superiority to conventional self-help, such as bibliotherapy, or WLT. There is currently one ongoing study registered with ISRCTN, comparing supported iCBT with day programmes for BN [19]. It is hoped that the results of this, in addition to further research, will provide more evidence. At present, however, we would be cautious in recommending such programmes as part of a treatment model.

## Additional files

**Additional file 1: Table S1.** The risk of bias in selected studies using The Cochrane Collaboration’s tool for systematic reviews [11]. The table shows the results of the full assessment of bias in each study, using the domains recommended by the Cochrane Collaboration.

**Additional file 2: Table S2.** Characteristics of studies. Table listing the studies included and identifying their study populations, inclusion/exclusion criteria, interventions, outcomes and follow-up periods.

**Additional file 3: Table S3.** Effect sizes comparing iCBT with control (WLT/bibliotherapy). The table shows calculated Hedges g effect sizes for each study, for each outcome given. The CIs are in brackets. Results in bold indicate statistical significance. Control II is bibliotherapy in Ruwaard et al. [15].

## Abbreviations

BAI: beck anxiety inventory
BAT: body attitude test
BDI: beck depression inventory
BMI: body mass index
BN: bulimia nervosa
CBT: cognitive behavioural therapy
CBTF2F: face-to-face cognitive behavioural therapy
CI: confidence interval
iCBT: internet-delivered cognitive behavioural therapy
DSM (IV/5): Diagnostic and Statistical Manual of Mental Disorders (4th/5th edition)
EAT-40: eating attitudes test
EDE (Q): Eating Disorder Examination (Questionnaire)
EDI (2): Eating Disorder Inventory (1991 revised edition)
EDQOL: Eating Disorders Quality of Life
F/U: follow up
HADS: Hospital Anxiety and Depression Scale
ITT: intention to treat
LOCF: last observation carried forward
NICE: The National Institute for Health and Care Excellence
RCT: randomized controlled trial
Rx: treatment
SCL-90 (R): Symptom Checklist 90 (revised edition)
SF-6D: short form six dimension
TCI-R: Temperament and Character Inventory (revised edition)
WHOQOL (BREF): The World Health Organisation Quality of Life (shortened version)
WLT: waiting list for treatment
