# Supplementary material for: The effectiveness of internet-delivered cognitive behavioural therapy for those with bulimic symptoms: a systematic review: A review of iCBT treatment for bulimic symptoms
Source: BMC Res Notes. 2018 Oct 22;11:748. doi: 10.1186/s13104-018-3843-2 (PMC6196450; doi:10.1186/s13104-018-3843-2)
Supplement: Supplementary file 1 — Additional file 1: Table S1. The risk of bias in selected studies using The Cochrane Collaboration’s tool for systematic reviews [11]. The table shows the results of the full assessment of bias in each study, using the domains recommended by the Cochrane Collaboration. [file 13104_2018_3843_MOESM1_ESM.docx]

Table S1: The risk of bias in selected studies using The Cochrane Collaboration’s tool for systematic reviews.^10^

| Domain | Support for Judgement | | | | |
| --- | --- | --- | --- | --- | --- |
|  | Sánchez-Ortiz et al.^14^ | Ruwaard et al.^15^ | Fernández-Aranda et al.^12^ | Wagner et al.^16^ | Zerwas et al.^13^ |
| Random sequence generation  (Selection bias) | Blinded statistician, stratified randomisation codes. | Computerised permuted block randomisation. No blinding. | No randomisation used. | True randomisation used from www.random.org. | Computerised permuted block randomisation used. Clinicians blinded. |
| **Review Authors’ Judgement** | **Low** | **Low** | **Unclear** | **Low** | **Low** |
| Allocation concealment  (Selection bias) | Sequence concealed until groups assigned. | Concealed from diagnosticians not research assistants. | No concealment noted. | Not mentioned. | Research coordinators blinded; therapists not blinded. |
| **Review Authors’ Judgement** | **Low** | **Unclear** | **Unclear** | **Unclear** | **Low** |
| Blinding of participants and personnel (Performance bias) | Participants and personnel not blinded. | Participants and personnel not blinded. | Participants and personnel not blinded. | Participants and personnel not blinded. | Participants and personnel not blinded. |
| **Review Authors’ Judgement** | **Unclear** | **Unclear** | **Unclear** | **Unclear** | **Unclear** |
| Blinding of outcome assessment (Detection bias) | Outcome assessors blinded to treatment allocation; 69% guessed. | Not blinded. All self- reported. | Outcome assessors not blinded. Self-report majority of outcome measures. | Not blinded. Outcome measures were assessed by 4 separate researchers all trained in “intensive rater training”. | Clinicians blinded. Some outcome measures self-reported. Primary and secondary outcomes assessed by independents. |
| **Review Authors’ Judgement** | **Unclear** | **High** | **High** | **Low** | **Low** |
| Incomplete outcome data  (Attrition bias) | All drop-outs recorded. ITT analysis used. No exclusions. | Adherence and attrition data reported. LOCF analysis used. | Total drop-outs mentioned. No mention of attempt to include these in analysis. | Drop-out rates reported; unclear whether used in analysis. No significant differences. | ITT analysis used. Dropout rates reported but felt not significant. |
| **Review Authors’ Judgement** | **Low** | **Low** | **Unclear** | **Low** | **Low** |
| Selective reporting  (Reporting bias) | All results reported. | All results reported. | No analytical comparison reported in groups pre and post treatment. | All results reported. | No analytical comparison reported in groups pre and post treatment. |
| **Review Authors’ Judgement** | **Low** | **Low** | **Unclear** | **Low** | **Unclear** |
| Other sources of bias | Recruitment of solely female students. | Nil significant. | Partial funding by the intervention manufacturers. | Nil significant. | Partial funding by pharmaceutical company. |
| **Review Authors’ Judgement** | **Unclear** | **N/A** | **High** | **N/A** | **Unclear** |
| **Overall Score** | **Low** | **Unclear** | **High** | **Low** | **Low** |
