# Supplementary figures and images for: The effectiveness of internet-delivered cognitive behavioural therapy for those with bulimic symptoms: a systematic review: A review of iCBT treatment for bulimic symptoms
Source: BMC Res Notes. 2018 Oct 22;11:748. doi: 10.1186/s13104-018-3843-2 (PMC6196450; doi:10.1186/s13104-018-3843-2)

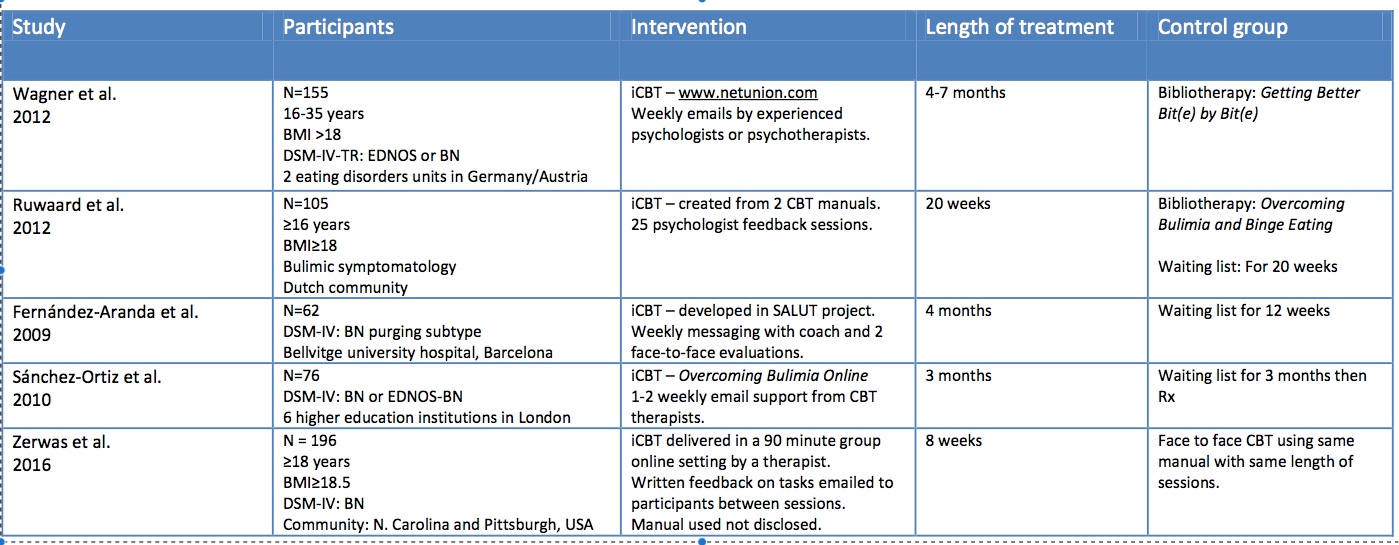
**Table S2: Characteristics of Studies**
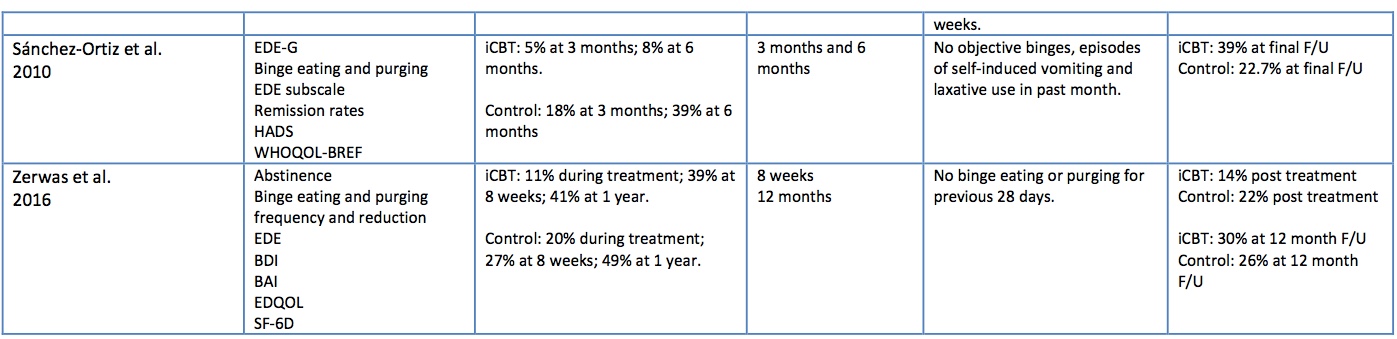

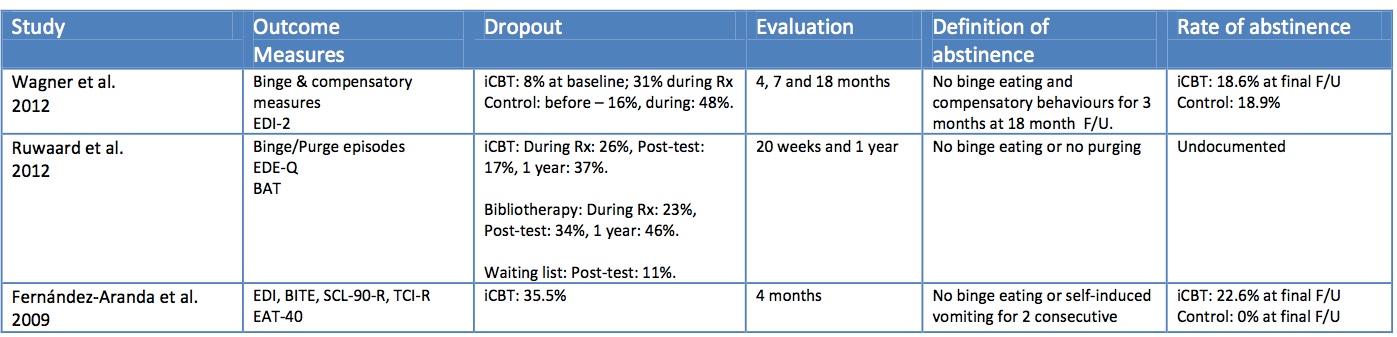

Supplement: Supplementary file 2 — Additional file 2: Table S2. Characteristics of studies. Table listing the studies included and identifying their study populations, inclusion/exclusion criteria, interventions, outcomes and follow-up periods. [file 13104_2018_3843_MOESM2_ESM.docx]
