# Supplementary material for: The effectiveness of internet-delivered cognitive behavioural therapy for those with bulimic symptoms: a systematic review: A review of iCBT treatment for bulimic symptoms
Source: BMC Res Notes. 2018 Oct 22;11:748. doi: 10.1186/s13104-018-3843-2 (PMC6196450; doi:10.1186/s13104-018-3843-2)
Supplement: Supplementary file 3 — Additional file 3: Table S3. Effect sizes comparing iCBT with control (WLT/bibliotherapy). The table shows calculated Hedges g effect sizes for each study, for each outcome given. The CIs are in brackets. Results in bold indicate statistical significance. Control II is bibliotherapy in Ruwaard et al. [15]. [file 13104_2018_3843_MOESM3_ESM.docx]

|  | | Wagner et al. (2012) | | | Ruwaard et al. (2012) | | Sánchez-Ortiz et al. (2010) | | Zerwas et al. (2016) | | Fernàndez-Aranda et al. (2009) |
| --- | --- | --- | --- | --- | --- | --- | --- | --- | --- | --- | --- |
|  | | Post Rx: 4m | 7m | 18m | Post Rx: 5m | 12m | Post Rx: 3m | 6m | Post Rx: 2m | 12m | Post Rx: 4m |
| Main Outcomes | | | | | | | | | | | |
| Binge eating | iCBT vs. control | 0.03  (-0.38 to 0.45) | -0.03  (-0.47 to 0.42) | -0.13  (-0.57 to 0.31) | 0.42 (0.90 to -0.05) | - | 0.40  (0.88 to -0.09) | -0.61  (0.04 to -1.16) | 0.06 (-0.24 to 0.35) | 0.09  (-0.2 to 0.39) | 0.48 (-0.52 to 6.73) |
|  | iCBT vs. control II |  |  |  | **0.85 (1.34 to 0.36)** | 0.34 (0.81 to -0.14) |  |  |  |  |  |
| Purging | iCBT vs. control | 0.17  (-0.25 to 0.58) | 0.17  (-0.28 to 0.61) | 0.17  (-0.27 to 0.61) | **0.88 (1.38 to 0.39)** | - | 0.3  (0.78 to -0.18) | **0.59**  **(1.14 to 0.03)** | 0.02 (-0.28 to 0.31) | 0.00 (-0.29 to 0.29) | - |
|  | iCBT vs. control II |  |  |  | **0.67 (1.15 to 0.19)** | 0.00 (-0.47 to 0.47) |  |  |  |  |  |
| Self-induced vomiting | iCBT vs. control | 0.14  (-0.27 to 0.56) | 0.14  (-0.3 to 0.59) | -0.04  (-0.47 to 0.4) | - | - | -0.33  (-0.81 to 0.16) | -0.4  (-0.95 to 0.15) | - | - | **0.77 (1.37 to 7.89)** |
| EDE score | iCBT vs. control | - | - | - | -0.5  (-0.97 to -0.02) |  | **1.23**  **(1.74 to 0.70)** | **0.97**  **(1.53 to 0.38)** | 0.00 (-0.29 to 0.29) | -0.06 (-0.36 to 0.23) | - |
|  | iCBT vs. control II |  |  |  | -0.37  (-0.84 to 0.11) | 0.14  (-0.33 to 0.61) |  |  |  |  |  |

**Table S3: Effect sizes comparing iCBT with control (WLT/bibliotherapy).**

***CIs are in brackets. Results in bold indicate statistical significance. Control II is bibliotherapy in Ruwaard et al. (2012).***
